# Book Review

**Published:** 1936

**Authors:** 


### Reviews of Books

**Some Thoughts of a Doctor.** By Frederick Parkes Weber, M.A., M.D. Pp. 183. London: H. K. Lewis & Co. Ltd. 1935. Price 6s.—The versatility, learning and wide range of this well-known writer, who has covered so much and so varied ground in the past forty years, has given us an interesting collection of essays. Within the brief space of less than 200 pages medical, psychological and moral issues are surveyed. Medically his speculations are fresh and stimulating, and the theory of the "safety valve" is full of interest, whilst his reflections upon rest and exercise and food are the well-ordered thoughts of maturity. Dr. Weber, in the psychological field, offers a balanced criticism of the Freudian thesis, and very fairly points to the riot of speculation which has tended to obscure the truth contained in the theories first put forward by Freud. In the moral field Dr. Weber's deductions from his principles will surely appear to most men as contrary to right reason. For having arrived at the position that man, having acquired a measure of control over the forces of Nature, he must use it for his good and for his good only, he fails to distinguish between what man is physically capable of and what he is morally entitled to do. Thus, whilst man can determine what things are for his good by examining the purposes of the various gifts and faculties with which God has endowed him, nevertheless to deprive himself or another of these faculties without just cause is clearly contrary to God's plan. Again, is it not important for clarity of thought on the subject to make the point that in individuals it is not the *amount* of free-will, but the power to exercise it which varies?

**Endocrine Tumours and other Essays.** By Frederick Parkes Weber, M.D., F.R.C.P., F.S.A. Pp. 207. London: H. K. Lewis & Co. 1936. Price 7s. 6d.—This small volume is intended as a companion to *Some Thoughts of a Doctor* published last year, though the subjects of the majority of the essays in this volume are more strictly medical. All are interesting, many are provocative of thought, and some provide sound medical teaching in a most palatable form. Their subject-matter bears tribute to the author's wide interests and deep learning. They range from early published descriptions of thrombo-angiitis obliterans and the probable effect of tight corsets in the etiology of chlorosis to the modern conceptions of endocrine neoplastic dysfunction and the pathology of splenic reticulosis. If there is one fault to be found with these essays it is the author's over-free use of parenthetical brackets, but even this will probably be regarded by those who know Parkes Weber as a characteristic exemplifying the inadequacy of the spoken or written word in the function of expression of his rapid flights of thought. The book will afford pleasure and stimulation to all, and will assuredly be acquired by many who know the author as a living memento of his great personality.

**A Synopsis of Physiology.** By A. RENDLE SHORT, M.D., F.R.C.S., and C. I. HAM, F.R.C.S. Second Edition. Edited by C. L. G. Pratt. Pp. vi., 312. Illustrated. Bristol: John Wright & Sons Ltd. 1936. Price 10s. 6d.—Excellent work has been done in bringing this useful little book up to date, and many improvements are included. It should now take its place in popularity beside its well-known companion volumes on Medicine and Surgery, for it is worthy of the position. The English is at times too familiar, and it is a pity that so many minor errors have been overlooked in the proof reading. Students will be alarmed at the size of their surface area if they use the formula given. The weight should, of course, be stated in kilograms not in grams, but the more widely-used nomogram deserves mention as well or perhaps instead of the formula. The use of italics is sometimes unfortunate. For instance, to say that "In *any* solution, product of H ions and OH ions is constant" only emphasises the lack of the word "aqueous" after "any"; and when it is decided in Chapter I that creatinine "is *not* formed from creatine in the body" it is inconsistent as well as pointless to revive the controversy in Chapter XI by quoting authors who say that it does occur and is a normal process. In the section on "Respiration" it is a little disappointing to see the retention of the obsolete term "infundibulum" and the mythical idea of an intrathoracic pressure that is normally negative. The latter, and what is described is more truly interpleural pressure, cannot exist until the space for it is artificially produced. But perhaps we are mistaken and partial double pneumothorax is a normal condition, as we infer from the information at the end of the same paragraph that "in newborn infants lungs completely fill chest cavity, and negative pressure is only gradually produced." Ideas of $CO_2$ transport should be modified to allow for the carbonic anhydrase theory for although it is mentioned no attempt is made to account for it. The explanation of Cheyne-Stokes Respiration is self-contradictory. Few will deny the necessity of mentioning some of the outstanding examples of experimental evidence, and the difficult task of selection and condensation has been on the whole well done, but occasionally the evidence offered is not above question. The three reasons given for the summary rejection of Cannon's theory of adrenaline action must be mentioned. The first is evidence that has, we think, not been repeated, but the second is widely regarded as unsound, and the third is a mere surmise. These criticisms, although by no means exhaustive, will not detract greatly from the value of this well-arranged book if it is used as its authors intended in conjunction with, or subsequent to, a normal medical course in Physiology.

**Diseases of Children.** By ROBERT HUTCHISON, M.D., LL.D., F.R.C.P. Seventh Edition. Pp. viii., 452. Illustrated. London: Edward Arnold. 1936. Price 21s.—Robert Hutchison's Lectures on Diseases of Children have served as an introduction to diseases of children to so many generations of students since the first edition appeared in 1904 that it scarcely needs an introduction to the majority of the profession. Since most of the chapters were delivered as lectures at the London Hospital, the whole work has a very pleasant colloquial flavour which makes it very easily read. In the preface the author disclaims any pretensions that this is a text-book of diseases of children; nevertheless, it deals in an essentially clinical manner with all the common disorders of infancy and childhood, and will be found sufficient for the majority of students. Even for those who later progress to the study of one of the larger "text-books" it will serve as an invaluable introduction to the subject. Questions of treatment are discussed in some detail, and there is an efficient index, so that the book should appeal strongly to every practitioner.

**Chronic Streptococcal Toxæmia and Rheumatism.** By J. D. HINDLEY-SMITH. Pp. xii., 276. London: H. K. Lewis & Co. 1935. Price 7s. 6d.—The author attempts to correlate the innumerable evidences of chronic toxæmia and of chronic rheumatism, with his conception of alkali depletion resulting from the progressive accumulation of bacteria, and of streptococci in particular, beyond the saturation point in susceptible individuals. In this he makes out a good case for his conception of a syndrome of chronic streptococcal toxæmia, and there is never any doubt of the honesty of his search for a rational treatment, both prophylactic and curative. There will, however, be much disagreement with his explanations of phenomena observed, and with the numerous unorthodox conceptions of the physiological and immunological processes by which he justifies many of his therapeutic procedures. The book is probably not intended as a scientific exposition of the streptococcal toxæmic theory of the causation of chronic rheumatism, but rather as a contribution to the practical handling of this disease. As such its study by the practitioner will provide him with many new view-points, and probably also with a more comprehensive picture of these manifestations of chronic ill-health than is usually obtainable from the more orthodox text-books of medicine.

**Reports on Chronic Rheumatic Diseases.** Edited by C. W. BUCKLEY, M.D., F.R.C.P. Pp. x., 172. Illustrated. London: H. K. Lewis & Co. Ltd. 1935. Price 12s. 6d.—This is the first annual report of the British Committee on Chronic Rheumatic Diseases appointed by the Royal College of Physicians, and consists of a number of original articles and critical commentaries on different aspects of this disease, each written by an authority on the particular subject under discussion. These articles are preceded by a report of the sub-committee on classification and nomenclature, and if the volume contained nothing more than this its value would still be assured. As is inevitable in any collection of papers of this type, the standard of medical value and of literary ability varies with the different authors, but every chapter bears the imprint of discriminating analysis and open-minded investigation. This report is concerned mainly with discussions of the various hypotheses of the causation of chronic rheumatism, and with some of its special manifestations.

Very little is said of treatment, a fuller discussion of this being promised in the next report. As an up-to-date and authoritative summary of our knowledge of some of the problems of chronic rheumatism this little volume will be welcomed by every practitioner in the country, and its presence on the shelves of consultants (medical, surgical and orthopædic) is almost inevitable. Illustrations are few but carefully selected, the paper is of good quality, the printing clear and pleasing, and the book is well indexed. In the last chapter is a complete list of all the important publications on chronic rheumatism from April, 1934, to March, 1935, subdivided into four three-monthly periods, and grouped according to the country of origin—an invaluable addition to research student and specialist alike.

**Slums and Slummers.** By C. R. A. MARTIN. Pp. viii., 185. Illustrated. London: John Bale, Sons & Danielsson. 1935. Price 6s.—This book is written by one who has obviously long and detailed experience of the slums. The author humbly states that his book is "an attempt to tell of what exists behind slumdom's foul front." The book certainly justifies the author's own conclusion that the present five-year slum clearance programme will not solve the problem in this country for ever. The book is nicely divided into six chapters: (1) Historical Survey, (2) and (3) Slums and Slum Dwellers, (4) Slum Clearance, (5) Re-housing, (6) Slums in the Making. Good as the book is, there is just a tendency throughout to a pessimism which has not affected all other workers in this field to this extent; nor will all workers agree that all illicit things are to be found in such abundance in the slums as this writer tends to suggest. Especially to those unacquainted with the slums the book can be heartily recommended.

**The Sanitary Inspector's Handbook.** By HENRY H. CLAY, F.R.San.I., F.I.S.E. Second Edition. Pp. xxii., 432. Illustrated. London: H. K. Lewis & Co. 1936. Price 15s.—That the first edition of this well-known manual was so soon exhausted, necessitating this second edition, has proved its value as the principal text-book, not only for students preparing for the sanitary inspector's examination, but as a reliable reference book for public health officers generally. The publication of the second edition provided the author with the opportunity of including appropriate legislation passed since then. This is noticeable in the chapter dealing with housing procedure, which has been entirely re-written in order to incorporate the Housing Act, 1935. The method adopted of giving a readable, concise summary of the law at the commencement of each section has been maintained and assists towards methodical study. The book, which has been extended by 46 pages, covers the entire groundwork, and though the variety of subjects dealt with are numerous, each section receives full and adequate treatment. The line drawings are of a good scale, very clear and distinct, and completely elucidate the text. There are 95 of these diagrammatic sketches, varying from the incidence of the sun's rays at various times of the year to those showing sections of the drainage systems of dwelling-houses, typifying the application of the building bye-laws relating to water closets, fittings, soil pipes, etc. The chapters on food inspection and fish inspection are well written, and the one on disinfestation is up to date concerning cyanide fumigation by local authorities in the attack on the bed-bug. The book contains 432 pages, is furnished with a full index, and can be well recommended.

**X-Ray Therapy.** By FFRANGCON ROBERTS, M.D., M.R.C.P. Pp. xii., 214. Illustrated. London: H. K. Lewis & Co. Ltd. 1936. Price 10s. 6d.—A good book, clearly and concisely written and well printed and illustrated. The subject-matter is well presented, and the book should be read not only by radio-therapists but also by the general surgeon, so that, as the author says, the X-ray Department may no longer be looked upon as the "Ante-room to the Post-Mortem Room." The many methods of treatment are mentioned, but one wishes that the author had given some idea of the results obtained by each method in the various lesions, so that one might form a better idea of which method to use. Some of the physics might, perhaps, have been dispensed with, since the practising radio-therapists, for whom this book is primarily intended, already possess this knowledge, but since these chapters are written in simple language they will, no doubt, be of great value to D.M.R.E. candidates. The general arrangement of the book is good, the bibliography full, the index adequate, and the book itself of a convenient size.

**Painful and Dangerous Diseases of the Ear.** By R. R. WOODS, F.R.C.S. Pp. x., 188. Illustrated. London: Oxford University Press. 1936. Price 15s.—A simpler title for this book would have been "Aural Inflammation," for only inflammatory conditions are included. The book opens with forty pages devoted to anatomy (physiology is not mentioned) and methods of examination. These are good, though somewhat unnecessarily detailed. The next section deals with diagnosis and treatment: great care is expended on the former, and it is very competently set out, but the latter is somewhat sketchy; for example, one and a quarter pages only are given to treatment of acute suppurative otitis media—and the author fails entirely to distinguish between "wet" (*i.e.* syringing) and "dry" (or hygroscopic) methods. We admire, however, the restraint he has observed in avoiding descriptions of operative technique on which space is frequently wasted in works of this nature; though in the last part of the book his interest in such details is not so well suppressed. The final fifty pages are devoted to intracranial complications, again very well described, if we admit that a certain degree of dogmatism must be allowed: it would be unwise to rely too implicitly on his description for the differential diagnosis of brain abscess, sinus thrombosis, etc., but naturally the practitioner would rarely accept such a responsibility unaided. The author is annoyingly inexact in his diction: he writes of "the drum" when he means "the membrane," of "the middle ear" when he means "the tympanic cavity," and of a "pulsating perforation," whatever that may be: "mastoidism" and "meningism" we encounter frequently, and always with the suspicion that he is not quite sure whether mastoiditis or meningitis is present or not. The book is excellently brought out, and profusely illustrated—indeed, the colour illustrations must excite wonder and envy equally: and is a full and competent exposition of the subject. To those general practitioners who require a book devoted to a somewhat limited field of surgery it can be thoroughly commended.

**A Practical Handbook of Midwifery and Gynæcology.** By W. F. T. HAULTAIN, O.B.E., M.B., B.Ch., F.R.C.S., F.C.O.G., and CLIFFORD KENNEDY, M.B., Ch.B., F.R.C.S., M.C.O.G. Second Edition. Pp. x., 356. Illustrated. Edinburgh: E. & S. Livingstone. 1935. Price 15s.—The first edition of this handbook was published nine years ago. During the intervening years so many advances have been made in midwifery and gynæcology—especially in the field of the endocrine control of the female generative organs—that numerous alterations have been necessary in the text of the second edition, and several new chapters have been added. A very large amount of information has been compressed into a comparatively small compass, and the book has been tabulated throughout, making it especially valuable to students preparing for examination. The authors have, as a rule, limited themselves to a statement of the most generally accepted opinion on any point that is controversial. They have given little space to discussing theories, nevertheless the subject-matter is up to date and in line with modern teaching. The chapters on the pathology of pregnancy and the complications of the puerperium are particularly good. The remaining nine chapters deal with gynæcology and the feeding of infants. Though the space allowed for these important subjects is somewhat limited, the authors give a concise and fairly complete account of the most important gynæcological diseases. The chapter on operative gynæcology is too short, and the operations described too few to bring it up to the standard of the rest of the text-book, and in the opinion of the reviewer it would have been better to omit it and to refer the reader to the larger text-books which specialize in this branch of operative surgery. This book can be strongly recommended to the student of medicine and to the practitioner who requires a short and up-to-date handbook on obstetrics and gynæcology.

**Insulin.** By DOUGLAS W. HILL, B.Sc., Ph.D., and FREDERICK O. HOWITT, M.Sc., Ph.D. Pp. xii., 219. Illustrated. London: Hutchinson & Co. 1936. Price 12s. 6d.—This volume, dealing with the work carried out on the isolation of insulin, its purification, and its physiological action, will be of the utmost value to scientists and physicians alike. The authors start the book with an historical account of the clinical and pathological researches which led to the conclusion that the pancreas is related to that insidious disease diabetes mellitus. As far back as 1686 Brunner suggested that the pancreas was in some way related to fat and carbohydrate metabolism; Opie and others later described the changes taking place in the islets of Langerhaus in diabetics. The clinical and pathological investigations were followed by attempts to isolate the principle concerned in regulating the metabolism of carbohydrates and fats ; numerous researches are described in which pancreatic extracts were made, and failed to relieve symptoms of diabetes when administered orally. Banting and Best, with their co-workers at Toronto, finally succeeded, by modifying a method introduced by Collip, in isolating an active principle by fractional precipitation of extracts with alcohol. It is perhaps invidious to make special reference to other workers, but it is undoubtedly true that H. W. Dudley, whose recent early death is much to be regretted, did much valuable work in this field of research. The mode of action of insulin is dealt with in Section 7, and various theories discussed. Biological methods of assay are also discussed in some detail. One of the outstanding features of this excellent volume is the extensive bibliography which it contains, and which will prove of great utility to those interested in the study of diabetes mellitus. The book has a local interest, as Dr. Hill is an old student of Bristol University. We hope that this volume will shortly be found in the library of all physicians and bio-chemists.

